# Supplementary material for: Are carbon emissions trading and green financial instruments synergistic? -Comprehensive quantitative research based on content analysis
Source: PLoS One. 2024 Mar 7;19(3):e0298601. doi: 10.1371/journal.pone.0298601 (PMC10919601; doi:10.1371/journal.pone.0298601)
Supplement: S1 Data — (ZIP) [file pone.0298601.s001.zip › Data/地级市Z_F工作报告.zip!/║╙─╧/╞╜╢Ñ╔╜╩╨/└┤╫╘╣1⁄2╓┌║┼í╛┬φ┐╦╩2╛▌═°í┐_2014.docx]

各位代表：

现在，我代表市人民政府，向大会报告工作，请予审议，并请各位政协委员和其他列席人士提出意见。

一、过去五年工作回顾

2013年，是本届政府任期届满之年，也是我市近年来经济社会发展形势最为严峻的一年。一年来，我们在省委、省政府和市委的正确领导下,在市人大、市政协的监督支持下，牢牢把握主题主线，围绕稳增长、调结构、破难题、惠民生，综合施策、攻坚克难，有效扭转了主要指标增速回落过大的局面，全市经济呈现出触底回升、稳定向好的发展态势。初步核算，全年生产总值增长6.6%；公共财政预算收入增长11.5%；固定资产投资增长22.6%；社会消费品零售总额增长14%；城镇居民人均可支配收入和农民人均纯收入分别实际增长6%和10.8%。

2013年工作目标的基本实现，标志着本届政府基本完成了市九届人大确定的各项目标任务。特别是经过不懈努力，我市被纳入全国老工业基地调整改造规划和资源型城市可持续发展规划等，平顶山加快发展的政策优势进一步凸显，全市上下凝心聚力、干事创业的氛围更加浓厚。

五年来，我们直面危机、多措并举，推动经济保持平稳发展。面对国际金融危机的严重冲击，从关键环节和紧迫任务入手，多措并举、长短结合，全力化解经济下行压力。把稳定工业经济运行作为首要任务，先后制定实施了工业发展腾飞工程、稳增长16条举措等一系列政策措施，深入开展企业大服务专项行动，加强银企对接、产销对接和煤电油气运调节，解决了一大批制约企业发展的突出问题，促进企业降低成本、开拓市场、释放产能。2013年全市规模以上工业增加值683.4亿元，与2008年相比，年均增长11.3%。把加强重点项目建设作为有力抓手，强化投资拉动，401个项目开工建设，239个项目建成投产，18个重大前期项目取得积极进展。累计完成固定资产投资4290.9亿元，年均增长25.3%。国家重点项目南水北调中线工程平顶山段全线贯通，干线征迁和移民安置工作受到国家和省表彰。把扩大消费作为重要举措，落实消费鼓励政策，推动城乡市场体系建设，实施“万村千乡市场工程”和“新网工程”，做好家电下乡、汽车摩托车下乡等工作，增强消费拉动。2013年社会消费品零售总额540.9亿元，年均增长17.1%。一系列应对危机的举措，有效推动了经济平稳增长。2013年全市生产总值1556.9亿元,年均增长9.1%;公共财政预算收入119.7亿元，年均增长13.9%。

五年来，我们构建载体、优化结构，推动发展方式加快转变。着力调优存量、扩大增量、弥补短板，加快产业转型升级。产业发展载体更加完善。10个省级产业集聚区累计完成投资1630亿元，入驻企业762家，规模以上工业主营业务收入达到839.3亿元。11个商务中心区和特色商业区发展规划全部获批，25个项目开工建设，累计完成投资52.7亿元。结构调整步伐更加坚实。实施高标准粮田“百千万”建设工程和现代农业产业化集群培育工程，新增土地流转面积62.6万亩、有效灌溉面积49.3万亩，建成高标准粮田42.5万亩，粮食连续10年丰收；新增省级农业产业化集群4个，培育现代循环畜牧业企业50家，新建改扩建标准化养殖园区1486个。实施产业转型升级“双千工程”、“双百工程”，改造提升传统优势产业，培育壮大战略性新兴产业，大力淘汰落后产能。平煤神马集团成功打通世界最完整的煤基尼龙化工产业链。六大高耗能行业比重下降3.1个百分点，装备制造、新型建材行业比重分别提高1.7个和5.5个百分点。实施现代服务业提升工程，编制完成旅游目的地总体发展规划，尧山—大佛景区晋升为国家5A级景区，并成为首批国家级生态旅游示范区，“中原大佛、神奇尧山，观音祖庭、近悦远来”旅游品牌日益彰显；广发银行、中信银行等14家金融机构入驻我市，平顶山银行实现跨区域经营和县域服务全覆盖，去年我市被评为全省金融生态环境优秀市；现代信息服务业快速发展。第三产业比重提高6.9个百分点。科技支撑作用更加突出。获国家科技进步奖9项、省科技进步奖53项，67项重大技术取得突破。新增院士工作站3家、博士后研发基地2家、国家级高新技术企业39家、省级工程技术研究中心29家。创建中国驰名商标7件、省名牌产品47个，专利授权4409件，成为全国科技进步先进市、国家知识产权试点市。生态建设成效更加明显。加强重点企业节能技术改造和公共机构节能，深化大气环境和白龟湖综合整治，万元生产总值能耗下降19%，市区空气质量优良天数和出境断面水质达到省定标准，城市集中饮用水源地水质达标率100%。新增造林85万亩，10个县（市、区）全部创建为省级林业生态县。

五年来，我们以创促建、强化引领，推动城乡一体协调发展。成功创建为国家园林城市、国家森林城市、省文明城市，国家卫生城市创建通过技术评估。城镇化率由40.2%提高到46.4%。规划体系逐步健全。城市新区建设总体方案获批，组团式发展规划编制完成，中心城区控制性详规实现全覆盖，市域城镇体系规划、6县（市）和石龙区城市总体规划完成报批，“一核三组三星”现代城镇体系框架初步形成。城市功能日益完善。按照新区加快建设、老城提档升级、新老城区同步发展的要求，统筹推进中心城区建设与管理。新城区累计完成投资172.9亿元，新增道路30公里、建筑面积150万平方米，建成区达到15平方公里，市博物馆、城市规划展示馆、文化艺术中心等标志性项目建成投用，现代化湖滨新城初具规模。老城区着力实施城中村开发和旧城改造，累计开工1156万平方米、主体完工928万平方米；新建改扩建道路318.7公里，延伸市政管网683公里。湖滨路、西南环路建成通车，解决了新老城区交通瓶颈制约。更新公交车386台。基本完成天然气置换人工煤气。城市精细化管理水平明显提升，社区物业管理实现全覆盖，市数字化城管系统投入运行，获全国数字城市建设示范市。县城和小城镇建设提质扩容。以快速通道建设为突破口，推动中心城区与鲁山、叶县、石龙组团式发展，推进汝州、舞钢、郏县向区域性中心城市发展。出台了加快中心镇建设的意见，引导人口、产业、生产要素向城镇集中。新增高速公路通车里程113.7公里，新建改建农村公路2591公里，实现了县县通高速、村村通公路。新农村建设扎实推进。启动建设中心村342个，建设新民居4.4万套，入住1.5万户，完成28万贫困人口脱贫，解决116.3万人饮水安全问题，农民生活居住条件明显改善。

五年来，我们迎难而上、敢于担当，推动改革攻坚持续深化。重点领域改革取得成效。政府机构改革、事业单位分类改革有序推进，市直机关所属企业脱钩改制基本到位。完成国企改制27家。平高集团与国家电网、普生药业与国药集团等实施战略重组或合作。卫生、文化、供销、林权等领域改革取得新进展。发展难题不断破解。理顺新城区、高新区管理体制，明晰化工产业集聚区市县共建共管责任主体，开展违法占地、违法建设集中整治，实施工业企业“退城进园”搬迁，完善市、区税费分成体制，完成农信社达标升级并启动组建平顶山农村商业银行，出台解决历史遗留房屋产权登记问题的具体办法，一批涉及全局和长远发展的急事、大事、难事取得阶段性成果或重大进展。对外开放进一步扩大。成功举办三届“华合论坛”，组团参加省内外重大招商活动，积极承接产业转移。2013年实际到位市外资金750亿元，利用外商直接投资4.5亿美元，年均分别增长22%和29.2%。与墨西哥圣路易斯市、巴西坎布里乌市结为友好城市。要素保障持续加强。大力开展农村土地和工矿废弃地综合整治，被确定为全国工矿废弃地复垦利用试点市，新增建设用地3.8万亩。成功发行15亿元城投债券，平煤神马财务公司开业运营，天瑞水泥在港交所上市。新增融资937.4亿元。培训各类技能人才48.3万人次。非公经济健康发展。新增各类市场主体3.3万户。2013年非公经济实现增加值809.6亿元，占生产总值的52%。

五年来，我们改善民生、创新治理，推动社会大局和谐稳定。坚持每年办好十项重点民生工程，解决了一批事关人民群众切身利益的问题。城镇居民人均可支配收入22482元,农民人均纯收入8541元,年均分别实际增长8%和11.1%。安全生产形势持续平稳。建立健全安全生产责任体系，全面推行安全生产网格化监管，积极推进企业安全生产风险预控管理体系建设，强力推进煤炭企业兼并重组，连续三年被评为安全生产优秀省辖市。就业和社会保障工作不断加强。新增城镇就业56.4万人，农村劳动力转移就业36.2万人，动态消除零就业家庭。全面落实城乡居民最低生活保障制度，实现动态管理下的应保尽保。持续提高城镇居民医保、新农合筹资标准和补助水平，城乡居民养老保险制度实现全覆盖，在全省率先建立社会临时救助制度。完善最低工资标准、退休人员基本养老金、城乡低保及农村“五保”供养标准正常调整机制，建立高龄老人及养老机构补贴制度。实施保障性安居工程项目130个，完成投资122.8亿元，保障家庭6.9万户。社会事业全面进步。学前教育三年行动计划圆满完成，新建改扩建幼儿园278所、中小学254所，城区幼儿入园难和中小学大班额问题得到缓解。职教攻坚计划顺利完成。高等教育办学水平明显提升，河南城建学院、平顶山学院通过教育部本科教学工作合格评估。深入实施文化惠民工程，建立覆盖城乡的公共文化服务体系。规划建设了宝丰文化改革发展试验区等特色文化园区，举办了全国戏剧梅花奖大赛、中国书法兰亭奖大赛、中国宝丰魔术文化节等大型文化活动，被授予中国书法城、中国观音文化之乡和中国汝窑陶瓷艺术之乡等称号。医疗卫生事业加快发展，17家公立医院全部实行药品零差率销售，政府办基层医疗卫生机构全部实施国家基本药物制度。全民健身运动广泛开展，竞技体育实现奥运金牌零的突破。人口计生工作继续保持全国先进。荣获全国残疾人工作示范市、无障碍建设先进市、民族团结进步模范集体。全国双拥模范城创建实现“四连冠”。老龄、妇女儿童、慈善、红十字等事业全面发展。社会大局和谐稳定。深化平安鹰城建设，创新群众工作机制，积极排查化解各类矛盾，信访形势总体平稳。加强食品药品监管和社会治安综合治理，人民群众安全感进一步提升。

五年来，我们转变作风、提高效能，推动政府自身建设不断加强。认真贯彻中央八项规定精神，严格执行国务院“约法三章”，扎实开展“树正气、转作风、促发展”集中教育实践活动，深入推进“两转两提”，大幅精简和规范行政审批事项，审批效能位居全省前列。自觉接受人大监督、政协民主监督和社会监督，认真办理人大代表建议和政协提案。加强法制宣传教育，被评为全国“六五”普法中期先进市。大力弘扬社会主义核心价值观，涌现出马俊欣、李博亚等一批先进典型。强化权力公开透明运行，完善惩治和预防腐败体系，廉政建设取得新成效。对台、统计、史志、盐业、气象、地震、烟草、档案、新闻出版、国家安全等工作都取得了新成绩。

回顾过去五年政府工作，我们深刻体会到：办好平顶山的事情，必须始终坚持科学发展，牢记发展第一要务，遵循发展规律，在保持经济平稳增长的同时，加快发展方式转变，努力实现速度、质量和效益相统一；必须始终加快调整转型，树立新型资源观，充分整合有形资源和无形资源，着力调结构、补短板、创优势，大力推进产业结构优化升级，坚定不移走好资源型城市可持续发展之路；必须始终注重改革创新，坚持解放思想不停步，以改革的思路和办法破解难题、扩大开放、创新驱动，借助外力，激活内力，增强发展动力；必须始终加强生态建设，把生态文明建设融入经济社会发展全过程，在保护中开发、在开发中保护，努力推动绿色循环低碳发展，让当代人受益，为子孙后代谋福祉；必须始终坚持为民惠民，把维护和实现最广大人民群众的根本利益作为执政之基，脚踏实地为群众办实事、做好事、解难事，真正做到发展为了人民、发展依靠人民、发展成果由人民共享；必须始终强化责任担当，坚持务实重干，敢于直面矛盾、动真碰硬，说了算、定了干、遇到困难不改变，一抓到底，努力创造经得起实践、人民和历史检验的实绩。

各位代表！

成绩来之不易，经验弥足珍贵。这是省委、省政府和市委正确领导的结果，是市人大、市政协积极支持的结果，是全市人民共同奋斗的结果。在此，我代表市人民政府，向辛勤工作在全市各条战线的广大干部群众，向关心、支持、参与平顶山建设发展的海内外朋友，表示衷心感谢并致以崇高的敬意！

在肯定成绩的同时，我们也清醒地认识到，受国际金融危机和资源型城市自身结构性问题影响，部分指标没有达到预期；传统支柱行业形势严峻，骨干企业生产经营还很困难；结构调整、转型发展任务艰巨，高新技术产业规模小、比重低，传统产业占比高，且多处于产业链前端和价值链低端，服务业发展薄弱；创新驱动能力不强，产学研用结合还不紧密；能耗水平偏高，雾霾等环境问题日益突出；城市基础设施建设欠账较多，解决好涉及群众切身利益的突出问题还需付出更多努力；政府职能转变还不到位，一些工作人员责任意识不强、履职能力不足、工作作风不实，行政服务效能还需提升，反腐倡廉工作需要进一步加强。对此，我们一定牢记使命、努力工作，采取有效措施，切实加以解决，决不辜负全市人民的期望！

二、今后五年目标任务

今后五年，是全面建成小康社会的决定性阶段，也是我市爬坡过坎、转型攻坚的关键时期。总的看，发展环境依然错综复杂，但也面临诸多有利条件和机遇。一方面，世界经济持续复苏的基础和动力不稳；我国经济增速放缓、有效需求不足的矛盾仍很突出；我市已进入资源型城市发展成熟期，传统资源优势逐步减弱，新的支撑力量尚待形成。另一方面，我国经济长期向好的基本面没有改变，全面深化改革将释放新的动力和活力；我市正处于工业化、城镇化加速发展阶段，国家把我市纳入全国老工业基地调整改造等规划，为争取更多项目和资金支持提供了有利条件。我们必须准确把握平顶山发展所处的阶段性特征，进一步增强机遇意识、忧患意识，以更大的勇气和智慧开拓前行，推动全市经济提质增效、行稳致远。

政府工作的总体要求是：以邓小平理论、“三个代表”重要思想、科学发展观为指导，全面贯彻落实党的十八大和十八届二中、三中全会精神，贯彻落实习近平总书记系列重要讲话精神，按照中央、省委和市委各项决策部署，以全面建成小康社会为目标，以调整结构、加快转型为主线，以提高质量和效益为中心，把改革开放贯穿于经济社会发展各个领域各个环节，更加注重优化经济结构，更加注重强化创新驱动，更加注重建设生态文明，更加注重保障和改善民生，转中求进，务实发展，坚定不移地走好资源型城市可持续发展之路，为中原经济区建设发挥重要战略支点作用。

今后五年的奋斗目标是：

——经济综合实力明显提升。生产总值年均增长9%以上，固定资产投资年均增长18%以上，社会消费品零售总额年均增长15%以上，公共财政预算收入年均增长10%，经济增长质量和效益大幅提升，中原经济区重要战略支点的地位进一步凸显。

——经济转型步伐明显加快。传统优势产业素质有效提升，支柱型接续替代产业进一步壮大，产业集聚集群效应更加突出，现代城镇体系基本健全。到2018年，能源、化工、装备制造、冶金建材、轻工食品等五大产业集群主营业务收入占工业比重达到90%以上，高新技术产业比重达到18%以上；第三产业占生产总值比重35%以上，非公经济比重55%以上；城镇化率55%左右。

——基础支撑条件明显改善。城市基础设施建设步伐加快，城乡一体化示范区框架基本形成，各类载体承载能力显著提升，电信网、广播电视网和互联网三网互联互通、融合发展的信息网络基本建成，现代综合交通体系更加完善，力争实现“北环过山、南环越湖、沙河复航、高铁通行、机场建成”，区域竞争新优势进一步增强。

——生态文明建设明显加强。资源节约型、环境友好型社会建设取得重大进展。循环经济发展水平和能源资源利用效率不断提高，单位生产总值能耗持续下降，主要污染物排放总量得到有效控制。到2018年，森林覆盖率达到33%以上，湛河等城市主要河道水质变清，白龟湖国家湿地公园建成，生态系统稳定性增强，美丽鹰城建设取得更大成效。

——发展内生动力明显增强。重点领域和关键环节改革取得重大进展，有利于科学发展的体制机制更加完善。开放型经济发展全面提速，对外开放深度和广度进一步拓展。全民受教育程度和人才培养程度普遍提高。自主创新体系更加健全，科技进步对经济增长的贡献率持续提升。

——人民生活水平明显提高。努力实现城乡居民收入和经济发展同步。基本公共服务均等化程度全面增强，就业更加充分，收入分配差距逐步缩小，住房保障体系基本形成。文化建设和民主法制建设取得新成效，公民思想道德素质和社会文明程度明显提升，社会更加和谐稳定。

各位代表！

上述目标任务，是时代赋予我们的庄严使命，是人民过上美好生活的热切期盼。我们坚信，只要不自甘落后、不消极等待、不畏惧困难，只要同心同德、提气鼓劲、加压奋进，只要加快赶、全力转、奋力超，完全可以摆脱被动局面，冲出经济低谷，创造美好未来。届时，一个经济振兴、文化繁荣、社会和谐、山川秀美、人民幸福的新鹰城，一定会展现在世人面前！

三、2014年重点工作

做好今年政府工作，要紧紧围绕全面建成小康社会目标，着力推进“转型提速、发展提质、环境提优、幸福提升”，努力在建设资源型城市可持续发展示范区、成为中原经济区重要战略支点的进程中，增创新优势、迈出新步伐。

经济社会发展的主要预期目标是：生产总值增长9%左右；公共财政预算收入增长10%；全社会固定资产投资增长21%；社会消费品零售总额增长14%；外贸出口总额增长15%；城镇居民人均可支配收入增长8%左右；农民人均纯收入增长9%左右；人口自然增长率控制在6.5‰以内；居民消费价格涨幅控制在3.5%左右；节能减排完成省定目标。

围绕上述要求和目标，着力做好十个方面工作：

（一）强化新型工业化主导，提升工业结构层次和发展水平。加快推进产业产品结构调整，全面增强工业竞争力。改造提升传统优势产业。利用现代技术改造提升煤炭、电力、化工、钢铁等传统优势产业，拉长完善产业链条，提高配套能力。发挥“退城进园”政策引导作用，推进神马帘子布东厂区等老城区工业企业向产业集聚区集中，进行“脱胎换骨”式改造，实现装备更新、产品换代、产业升级。积极培育战略性新兴产业。围绕培育壮大生物医药、节能环保、新能源、新材料等战略性新兴产业，实施圣光医药孵化园三期、武汉凯迪生物质合成油等52个重大项目。加快发展高成长性制造业。围绕发展装备制造、电子信息、轻工食品等高成长性制造业，重点抓好隆鑫产业园、尧谷光电等100个重大项目建设。加强工业经济运行调节。继续实施企业大服务专项行动，开展百家企业集团培育工程，做好煤电油气运供需衔接，确保生产稳定运行。

调整产业产品结构最终要落实到培育产业集群上，结合我市实际，着力打造能源、化工、装备制造、冶金建材、轻工食品等五大产业集群。能源产业集群，重点打造煤炭、电力、新能源三大产业板块，主营业务收入增长8%以上。化工产业集群，重点打造煤焦化工、盐化工、尼龙化工三大产业板块，主营业务收入增长10%以上。装备制造产业集群，重点打造高端装备制造、成套设备、核心基础零部件、三轮机车四大产业板块，主营业务收入增长13%以上。冶金建材产业集群，重点打造钢铁、有色金属、建材三大产业板块，主营业务收入增长9%以上。轻工食品产业集群，重点打造轻工、纺织服装、食品、医药及医疗器械四大产业板块，主营业务收入增长15%以上。实现上述目标，必须强化产业集聚区建设。完善产业配套，理顺管理体制，加快产业集聚区与所在行政区套合。10个省级产业集聚区投资增长21%左右，规模以上工业主营业务收入增长15%左右。

（二）强化现代服务业培育，提升第三产业规模和水平。以商务中心区和特色商业区建设为载体，着力培育现代物流、文化旅游、金融保险、商贸流通、信息服务等五大板块，确保第三产业增速高于经济增速。现代物流业。加快建设市现代物流产业集聚区和平西、平南物流园区，努力打造豫中南区域物流中心。在提升传统物流、发展第三方物流的同时，着力培育第四方物流。文化旅游业。实施尧山—大佛景区提升改造工程，推进尧山机场、311国道改线、大型停车场等旅游基础设施建设，抓好白龟湖国家湿地公园、香山文化旅游产业园等项目建设，深化旅游管理体制改革，实施旅游标准化和智慧化工程，发展乡村旅游，加快建设海内外知名的旅游目的地。金融保险业。加快组建平顶山农村商业银行，支持舞钢、叶县村镇银行组建工作，新引进2-3家金融机构。推进直接融资性资本市场业务发展。防范和化解金融风险。新增融资216亿元以上。商贸流通业。引入现代管理理念和新型商业模式，鼓励大型商贸企业建设网上商城和中小微企业开设网店；推进建业十八城、新华园怡购城、鹰城国际商务（商贸）中心等项目建设，着力培育一批年销售收入超亿元的大型商贸流通企业、税收超千万元的楼宇经济和城市综合体。信息服务业。抓住国家支持信息消费的机遇，以云计算、物联网、移动互联网为重点，培育发展新兴信息服务业态。同时，推动房地产业持续健康发展，抓好科技研发、工业设计、教育培训、健康服务、养老及家庭服务等新兴服务业的培育和发展。

（三）强化农业基础地位，提升现代农业发展水平。以稳定粮食生产、促进农民增收为核心，全面落实强农惠农富农政策，着力构建现代农业产业体系。推进种植规模化。引导承包地经营权有序流转，发展多种形式的适度规模经营，新增土地流转面积10万亩以上。继续实施高标准粮田“百千万”建设工程，创建高标准粮田20万亩，确保粮食总产稳定在200万吨以上。启动实施都市生态农业工程，规划建设一批兼具城市“菜篮子”、生态绿化、休闲观光等功能的现代农业园区。推进养殖标准化。开展养殖标准化示范创建活动，加快生态循环畜牧业试验示范市建设，大力发展生猪、肉牛、奶牛等三大产业集群，新建、改扩建标准化养殖场110个，新增大型循环畜牧业试点企业15家。推进经营产业化。继续实施现代农业产业化集群培育工程，着力发展具有地方特色的农业产业化集群。坚持家庭经营基础性地位，培育壮大专业大户、家庭农场、农民合作社、农业企业等新型农业经营主体，鼓励和引导工商资本到农村发展适合企业化经营的现代种养业，新增农民合作社300家，市级以上农业产业化龙头企业达到210家。推进生产机械化。落实大型农机购置补贴政策，新增农机总动力6万千瓦，主要农作物生产基本实现全程机械化。同时，大力加强农田水利基础设施建设，重点抓好北汝河治理、南水北调平顶山段配套工程等项目，新增有效灌溉面积8万亩；完善农畜产品质量监管监测体系，推广农业先进适用技术，提高气象现代化水平，确保农产品产量增加、品质提升。

（四）强化新型城镇化引领，提升城乡统筹发展水平。以人的城镇化为核心，科学推进新型城镇化建设，城镇化率提高1.8个百分点。加快农业转移人口市民化。坚持“一基本两牵动”，着力促进城镇基本公共服务常住人口全覆盖，重点解决一批已进城就业定居的农民工落户，成建制转化一批城镇棚户区和城中村居民，有序转移一批农村富余劳动力。切实保障进城农民土地承包经营权、农户宅基地用益物权和农民集体经济收益分配权，消除农民进城的后顾之忧。完善城镇规划体系。加快编制城乡一体化示范区控制性详细规划，完成北部矿区综合整治总体规划和沙河生态景观带规划，推进地下空间规划控制和综合利用工作。增强城镇综合承载能力。加快老城区提档升级，抓好平安大道西延、新南环路东延、开发一路北延等项目建设，推进北部矿区整治和湛南新城建设，城中村开发和旧城改造开工220万平方米、主体完工200万平方米。全面推进城乡一体化示范区建设，实施东部路网构建、西部“五横四纵”道路建设和焦庄水厂供配水工程，加快未来路、紫金山路、吉祥路建设，实现新老城区交通顺畅。完善热力管网，完成姚电公司两台机组供热改造。建设改造新升、郑营等13个农产品市场。加快宝石、平叶、平郏、平鲁等快速通道建设，实现中心城区与组团间和卫星城的快速连通。严格实施制止违法占地、违法建设暂行办法，提高城市精细化管理水平。巩固国家森林城市、国家园林城市等各项创建成果，确保国家卫生城市创建成功。继续抓好县城和中心镇建设，开展园林乡（镇）创建活动。支持石龙区独立工矿区改造搬迁项目建设。分类推进新农村建设。对城中村、城郊村、产业集聚区内和乡镇政府所在地的村庄，按城市社区标准进行规划建设；基本建成和初具规模的中心村，完善配套设施，引导搬迁入住，加快旧址复耕；已经开工建设的进一步论证，充分尊重乡村和农民意见，针对不同情况进行规范，防止出现“半拉子”工程；未启动建设的中心村，按照“五规合一”要求，做好规划，重新报批。加快农村基础设施建设，新建改建农村公路240公里，新增沼气用户2500户，新解决30万人饮水安全问题。

（五）强化重点项目支撑，提升经济社会发展后劲。大力实施项目工作法，突出抓好“三个重大”项目建设，确保年度完成投资800亿元以上。重大基础设施项目。抓好武西高速尧山至栾川段、孟平铁路复线、郑万高铁、沙河复航等100个项目，总投资647亿元，年度计划投资130亿元。重大经济发展项目。抓好平煤神马集团25万吨己二酸和20万吨己内酰胺、化工产业集聚区2×35万千瓦热电联供机组、宝丰不锈钢产业园等200个项目，总投资2535亿元，年度计划投资573亿元。重大民生工程项目。抓好市四十一中移址重建、新区人民医院、国开行融资的4个棚户区改造等100个项目，总投资530亿元，年度计划投资105亿元。“三个重大”项目建设是我市转型发展的重要基础和有力支撑，我们将制定专项行动计划，实行模拟审批，健全推进机制，营造良好环境，力争早日建成！

（六）强化创新体系建设，提升发展驱动力。深入实施创新驱动战略，增强科技创新引领和支撑作用。着力提升创新能力。强化企业创新主体地位，鼓励企业设立研发机构，培育省级工程技术研究中心5家、市级研发中心20家、产业技术创新联盟5个。深化与郑州大学、平煤神马集团三方战略合作，加快与清华大学组建煤基化工联合实验室、与上海交通大学共建产业技术研究院。支持平高集团、圣光集团建立海外研究机构，建成国家高压电器产品质量监督检验中心。大力发展科技型中小企业，培育国家级高新技术企业6家，高技术产业增加值增长15%以上。加快提升信息化水平。实施“宽带平顶山”工程，开展光纤到户、4G移动通信网等先进网络建设。促进工业化与信息化深度融合，加快“智慧城市”和农村信息化建设，大力发展电子政务，推动民生和社会领域信息化应用。加强人才队伍建设。深入实施鹰城人才计划、全民技能振兴工程和职教攻坚二期工程，完成各类职业技能培训12万人次。完善有利于创新的体制机制，保护和调动创新型人才积极性。

（七）强化文化强市建设，提升文化软实力。充分挖掘文化资源，加快文化改革创新，用文化滋养城市、引领风尚、推动发展。大力发展文化事业。继续实施农村和学校公益电影放映、舞台艺术送农民等文化惠民工程，推进广播电视“户户通”工程，深入开展群众性文化活动。做好重要文化遗产保护，加快矿山遗址公园等项目建设，推进国家级（宝丰）说唱文化生态保护实验区建设。广泛开展全民健身运动，抓好省级全民健身示范市创建工作。加快发展文化产业。落实扶持政策，推动数字传媒、演艺娱乐、文化旅游、工艺美术等产业做大做强，抓好舞钢影视文化产业园、宝丰文化创意产业园等项目建设。完善文化管理体制，推进公益性文化事业单位内部改革，完成平顶山日报报业传媒集团组建工作，加快文化影视传媒集团、广播电视传媒集团组建步伐。积极倡导文明新风。培育和践行社会主义核心价值观，弘扬“开放、包容、务实、创新”的平顶山精神。以创建全国文明城市为载体，持续开展“讲文明、树新风”主题实践活动，不断提高城乡文明程度。深入推进诚信建设和公民道德建设，广泛开展志愿服务活动，发展健康向上的网络文化，增强社会正能量。

（八）强化生态文明建设，提升可持续发展能力。把生态文明建设摆在更加突出的地位，加快打造天蓝、地绿、水净的美丽鹰城。进一步改善环境质量。实施蓝天工程，积极推进重点行业脱硫脱硝除尘改造、燃煤锅炉集中整治、机动车尾气治理、建筑施工和道路扬尘防治、餐饮油烟和秸秆焚烧整治，有效遏制雾霾污染。实施碧水工程，加快湛河综合治理及城乡沟河连通，推进新污水处理厂建设及老厂升级改造、白龟湖饮用水源地生态保护、白鹭洲湿地公园提升改造等项目，改善水环境质量。实施乡村清洁工程，抓好城市周边和农村面源污染治理及环境连片综合整治，建设美丽乡村。提高资源集约节约利用水平。大力发展循环经济，全面实施节能降耗行动计划，开展重点行业能效对标达标活动。加强再生资源回收网络体系建设，工业固体废弃物综合利用率达到91.5%。积极推广清洁能源。落实最严格的水资源管理制度，坚守“三条红线”，从严考核奖惩，加快节水型社会建设。提升生态自然修复能力。继续实施林业生态建设提升工程，加强水源涵养林、水土保持林、农田防护林和南水北调工程绿色观光产业带建设，完成造林8万亩、森林抚育10万亩。加快煤矿沉陷区治理和工矿废弃地整治。构建生态文明长效机制。深化主要污染物排放总量预算管理，完善污染物排放许可证制度，建立项目环评审批与当地容量挂钩机制。实行最严格的源头保护、损害赔偿和责任追究制度。

（九）强化改革攻坚和开放带动，提升发展动力和活力。加快实施一批看得准、有条件、有权限的改革，努力推动重要领域和关键环节改革取得新进展。推进行政体制改革。全面完成政府机构改革任务，加快食品药品监管、卫生和人口计划生育等部门职责和机构整合；调整工商、质监管理体制，实行分级管理；推动交通运输管理体制改革。加快事业单位分类改革。推进工商登记制度改革，逐步由先证后照改为先照后证。深化市场主体改革。加快中房公司、燃气总公司等企业改革，完成星峰集团、人民商场、达昌机械厂等企业破产终结。积极发展混合所有制经济，支持非国有资本参股国有资本投资项目。大力发展非公有制经济，引导关闭整合后的小煤矿、小焦化等民营企业就地实现转型发展，支持各类市场主体进一步做大做强。加快要素保障机制改革。实施全面规范、公开透明的预算制度；落实国家“营改增”、资源税从价计征等政策；盘活财政存量资金；加强政府债务管理；将市级个人所得税等四项税种下划市内三区。深化“人地挂钩”试点工作；探索实行租让结合、分阶段出让的工业用地供应制度；加强土地整治，节约集约用地。推进煤电一体化发展，加快实施大用户直购电试点和煤电价格联动机制，基本完成农电体制改革。深化户籍制度改革。有序放开中心城区落户限制，全面放开县城和建制镇落户限制，逐步提高户籍人口城镇化水平。探索推行居住证制度，建立以居住证为依据的基本公共服务提供机制。积极推进农村改革。坚持和完善农村基本经营制度，赋予农民更多财产权利。加快土地承包经营权、农村集体建设用地及宅基地使用权确权登记颁证工作。在符合规划和用途管制前提下，探索农村集体经营性建设用地进入市场交易办法。深化供销合作社改革。

大力实施开放带动战略，以开放促改革、促转型、促发展。拓宽开放领域。加快服务业、农业、基础设施和社会事业等领域开放，力争在银行保险、现代物流、文化旅游、科技、医疗、教育等领域引进一批合作项目。持续开放招商。围绕主导产业，绘制产业图谱，开展产业链招商和定点定向招商；发挥企业在招商引资中的主体作用和商会协会的桥梁作用，开展以商招商和中介招商。推行“二分之一”工作法，坚持大员上前线，多方配合联动，强化激励约束，努力形成大招商、招大商的浓厚氛围。全力办好第五届“华合论坛”，组织好珠三角、长三角等专题招商活动，全年实际利用市外资金790亿元。同时，优化出口结构，扩大出口规模，发展服务贸易。优化开放环境。启动筹建海关、检验检疫派出机构等工作，完善招商引资信息公共服务体系、外来客商投诉综合服务平台。牢固树立纳税人至上的观点，切实做好服务企业工作，对损害发展环境的案（事）件，发现一起，严查一起，绝不姑息！

（十）强化民生改善和社会治理，提升和谐社会建设水平。继续办好十项重点民生工程，让人民群众得到更多实惠。促进城乡居民增收。深化收入分配制度改革，完善机关事业单位工资和津补贴制度，推行企业工资集体协商，适时调整最低工资标准，提高农民经营性收入和在土地增值收益中的分配比例，多渠道增加低收入者收入，扩大中等收入者比重，提高消费能力。推动实现高质量就业。实施更加积极的就业政策，加大就业困难群体帮扶力度，重点做好高校毕业生、农村转移劳动力、化解产能过剩中出现的下岗失业人员就业工作。全年城镇新增就业7万人，农村劳动力转移就业5万人。加快完善社会保障体系。提高企业退休人员基本养老金、城乡居民低保标准，落实城镇职工基本养老保险与城乡居民养老保险衔接办法，推动机关事业单位养老保险制度改革；巩固全民基本医保，通过改革整合城乡居民基本医疗保险制度；加快实施社会保障“一卡通”。城镇居民医保和新农合人均补助标准提高到320元，重大疾病补偿比例提高到70%左右，全面实施大病保险。抓好市流浪未成年人救助保护中心、残疾人康复中心、夕阳红老年公寓扩建等项目建设，支持慈善事业发展。加快保障性安居工程建设，新开工保障性住房1.47万套、基本建成1.19万套。加大老区建设和扶贫开发力度，再实现4.8万人稳定脱贫。全面发展社会事业。启动二期学前教育三年行动计划，加快中小学布局调整，推进职教园区建设，提升高等教育办学水平。深化医药卫生体制改革，巩固完善基本药物制度。开工建设市儿童医院、2个全科医生临床培养基地、2个县级妇幼保健院，改扩建15个乡镇卫生院，升级改造174个村卫生室。继续实施社会办医行动计划。着力构建和谐医患关系。坚持计划生育基本国策，调整完善生育政策，促进人口长期均衡发展。持续抓好安全生产。深入开展安全生产网格化监管，全面推进企业安全生产风险预控管理体系建设，大力实施全民安全素质提升工程，坚决遏制重特大事故发生。加强和创新社会治理。坚持依法治市，建设法治鹰城。持续推进平安建设，完善应急管理体系，加强社会治安综合治理，依法打击违法犯罪活动。抓好社区建设，争创全国和谐社区示范区。做好第八届村委会换届选举。改革信访工作制度，畅通信访渠道，及时就地化解社会矛盾。强化食品药品安全监管，坚决治理餐桌上的污染，确保人民群众“舌尖上的安全”！

继续加强新形势下国防教育和国防后备力量建设，支持驻平解放军、武警部队、预备役部队、民兵和人民防空建设，促进军民融合深度发展。深入开展双拥共建，巩固发展军政军民团结。支持工会、共青团、妇联等人民团体发挥桥梁纽带作用。加强统计工作，搞好第三次全国经济普查。切实做好外事、侨务、对台工作。继续做好民族、宗教、史志、测绘、地震等工作。

四、切实加强政府自身建设

打铁还需自身硬。面对发展新形势，面对人民新期待，必须大力学习弘扬焦裕禄精神，扎实开展党的群众路线教育实践活动，以改革创新精神推进政府自身建设，真正做到为民务实清廉。

（一）切实加强学习，提升履职能力。持续实施公务员素质提升工程，加大对新知识、新技术的学习培训，增长学识、提高本领。加强调查研究，善于把握和运用市场经济规律，抓住工作重点，调动各方力量，敢做善成，提高应对复杂局面的能力。加强和改进公务员管理，完善考试录用、交流轮岗、竞争上岗等制度，健全创先争优长效机制，着力建设高素质公务员队伍。

（二）转变政府职能，提高行政效率。开展“服务质量提升年”活动。进一步简政放权，继续清理行政审批事项，对保留的审批事项建立权力清单制度，清单之外的一律不得实施审批。强化政府公共服务、市场监管、社会管理、环境保护等职责，推动政府职能转变。加强政府绩效管理，强化行政问效，坚决整治不作为、慢作为、乱作为等行为，切实提升政府执行力。

（三）推进依法行政，自觉接受监督。严格按照法定权限和程序行使权力、履行职责，推进政府工作法治化。坚决执行人大及其常委会的决议决定，自觉接受法律监督和工作监督，主动接受政协民主监督，认真办理人大代表建议和政协提案，广泛听取民主党派、工商联、无党派人士和人民团体意见。强化行政监察和审计监督，深化政务公开，接受社会监督。加强政府法制工作，改革行政执法体制。完善重大决策合法性审查和风险评估机制，提高政府决策民主化、科学化水平。

（四）强化责任担当，勇破发展难题。改革已进入深水区和攻坚期，我们必须进一步解放思想，牢固树立改革意识、担当意识，坚决消除阻碍发展的思维定势，坚决突破利益固化的藩篱，坚决革除体制机制的弊端，特别是对久攻不下、迟迟没有进展的疑难问题，敢于涉险滩，敢啃“硬骨头”，用创新性举措扫除发展障碍，让改革红利充分释放，让创造潜能充分迸发，让公平正义充分彰显！

（五）加快作风转变，坚持廉洁从政。严格落实中央八项规定精神、国务院“约法三章”和省委、市委意见，切实做到“三严三实”，持续解决“四风”问题。厉行勤俭节约，反对铺张浪费，严格控制一般性支出，真正把有限的财力用在推动发展和改善民生上。不断加强惩治和预防腐败体系建设，严格遵守廉洁从政的各项规定，努力实现干部清正、政府清廉，做人民满意的公仆。

各位代表！

新的征程已经开启，新的目标催人奋进。让我们在省委、省政府和市委的坚强领导下，用信心凝聚力量，用实干谱写华章，砥砺奋进，开拓创新，为全面建成小康社会、加快建设美丽鹰城而努力奋斗！
